# Acanthomatous Ameloblastoma: An Early Stage Case Report with Difficult Management

**DOI:** 10.1155/2021/9941779

**Published:** 2021-06-18

**Authors:** Roberto Pippi, Marcello Santoro, Alessandra Pietrantoni, Angelina Pernazza

**Affiliations:** ^1^Department of Odontostomatological and Maxillo Facial Sciences, Sapienza University of Rome, Via Caserta 6, 00161 Rome, Italy; ^2^Department of Molecular Medicine, Sapienza University of Rome, Italy

## Abstract

A case of a mandibular acanthomatous ameloblastoma, with an admixed little component of follicular type, is reported. The epidemiological features of the present case agree with those reported in the international literature. Clinico-radiographic differential diagnostic difficulties existed with several other noncystic osteolytic lesions of the mandible. Recurrence was diagnosed early 1 year after the initial excisional biopsy, and a definitive rim resection was therefore performed. No further recurrence occurred in the following 4-year follow-up.

## 1. Introduction

Ameloblastoma belongs to the benign epithelial odontogenic tumor subgroup, and it is probably one of the most controversial and enigmatic tumors of the facial skeleton, due to both clinical behavior and histological features [1–10].

The World Health Organization in 2005 [8] defined four variants: solid/multicystic (11-90%), unicystic (5-22%), desmoplastic (0.9-13%), and extraosseous/peripheral (0.5-9.3%) [5, 8, 9]; although in 2017 [11], the desmoplastic variant was included in the solid/multicystic forms, under the common term of ameloblastoma (9310/0 ICD-O code).

Unlike unicystic, peripheral, and desmoplastic types, which show a good response to the lowly invasive local excision, the solid/multicystic variant are more infiltrative with a high rate of local recurrence, especially following nonaggressive procedures, such as enucleation and curettage [1, 4, 6, 7].

The term acanthomatous is used in the presence of extensive squamous metaplasia and variable keratinization of stellate reticulum-like cells; in addition, the formation of squamous edges, in the center of the neoplastic nests, and calcification may be present. Acanthomatous ameloblastoma (AA) also presents histological features very similar to those of the squamous odontogenic tumor from which it differs since the peripheral cells are columnar instead of flat [9]. In an extensive review of 3,677 cases performed by Reichart et al. in 1995 [1], the AA was the third most frequent histological variant (12.1%) after the follicular (32.5%) and plexiform (28.2%) ones, although various histological aspects may be present in each ameloblastoma [11]. Very similar data were found in a multicenter study performed by Dhanuthai et al. [5] with a sample of 1,289 North American and Asian cases, on the basis of the 2005 WHO classification [8], with an incidence rate of 7.06% for the AA and 27.7% and 21.1% for the follicular and plexiform forms, respectively.

## 2. Case Presentation

**Figure 1:**
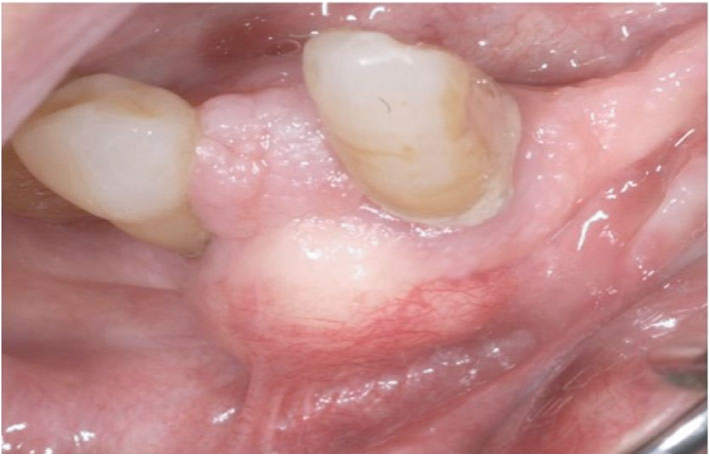
First visit clinical view shows a localized swelling at the right inferior alveolar process.

**Figure 2:**
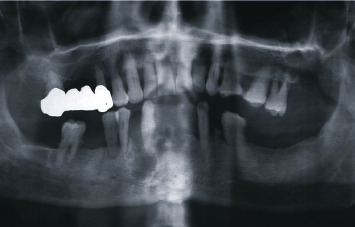
Orthopantomography at first visit showed a homogeneous, radiolucent, oval-shaped, unilocular area, which appeared quite well-delimited by a peripheral radiopaque and slightly scalloped border.

A 78-year-old patient was first observed in December 2008 at the Complex Operative Unit of Oral Surgery at the Department of Odontostomatological and Maxillofacial Sciences of the “Sapienza” University of Rome due to a small, symptomless, localized swelling at the right inferior alveolar process, between the canine and the first premolar which appeared spaced several millimeters each other (Figure 1). The swelling was reported to appear a few months earlier and was hard fibrous and painless on palpation. The contiguous teeth were firm and vital to the cold test, and neither the involvement of the lingual aspect of the mandible nor of the regional lymph nodes was detected on clinical examination. The patient was suffering from hemiparesis caused by cerebral ischemia in 2007, from noninsulin-dependent diabetes and from polio disability of the lower legs since he was 12 years old. The orthopantomography showed a homogeneous, radiolucent, oval-shaped, unilocular area, well-delimited by a peripheral radiopaque and slightly scalloped border, involving the roots of the canine and the first premolar and vertically oriented, from the top of the alveolar process up to about 1 cm below the apices of the neighboring teeth (Figure 2).

The en bloc excision of the lesion was directly performed under local anesthesia and after antibiotic prophylactic regimen (amoxicillin+clavulanic acid, 2 gr p. os, 1 hour before surgery) involving the overlying keratinized tissue and the 2 contiguous teeth, with a few millimeters of lateral and apical clinically healthy tissue and about 2 mm of residual bone cavity curettage. A surgical dressing was finally placed to support a secondary intention healing. No medical treatment was prescribed after surgery but only the use of a 0,20% chlorexidine mouthwash and analgesics as needed.

An acanthomatous ameloblastoma (AA), with a little component of follicular histo-type, was histologically diagnosed, and a microscopic amount of pathological tissue was identified at the deep edge of the surgical piece corresponding to the center of the residual bone cavity, while the lesion appeared completely removed from the contiguous soft and bone tissues.

**Figure 3:**
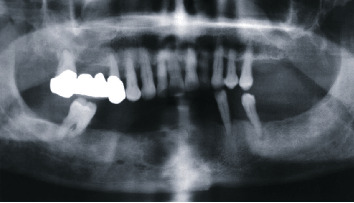
Orthopantomography 1 year after initial treatment: a radiolucent area was detected in the middle of the mineralizing bone tissue.

A close clinical radiographic follow-up was therefore performed until, after approximately 1 year, a 5 mm radiolucent area was detected in the middle of the mineralized bone tissue (Figure 3).

**Figure 4:**
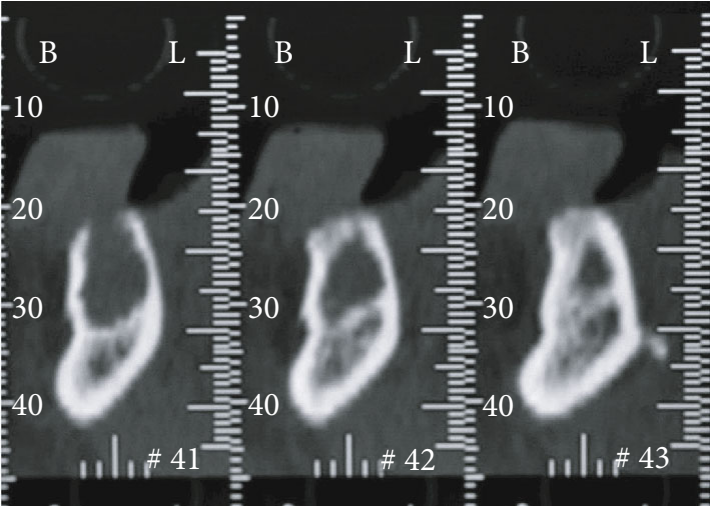
Dentascan CT: cross sections detail showed the limited involvement of the lingual cortical bone the limits of the recurrence.

A computed tomography (CT) with the Dentascan program was then performed (Figure 4) to better evaluate the morphology and the limits of the possible recurrence, and an incisional biopsy was performed to obtain microscopic confirmation.

**Figure 5:**
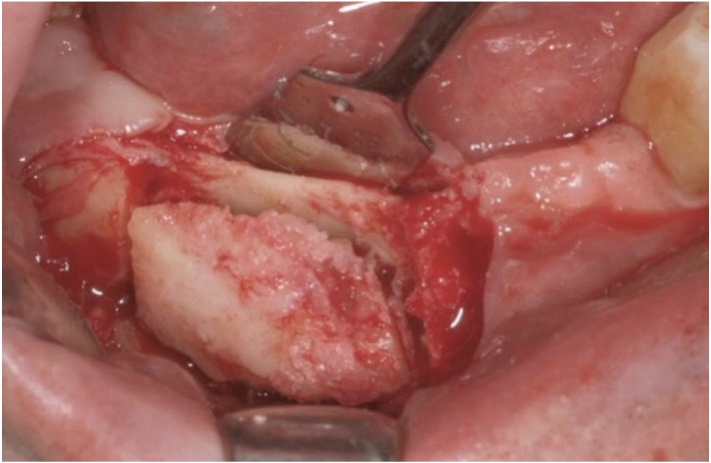
Rim resection intraoperative view.

Complete excision of the lesion was then performed by a rim resection involving a safety margin of about 7-8 mm of laterally and inferiorly radiographically healthy bone tissue, while preserving the integrity of the lingual cortical bone (Figure 5).

**Figure 6:**
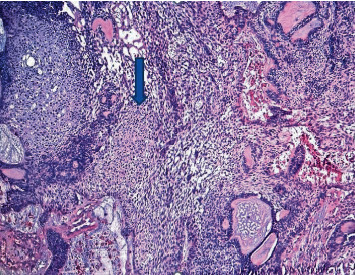
Histological preparation from the definitive surgically excised tissue, evidence of squamoid area (arrow), H&E ×4.

**Figure 7:**
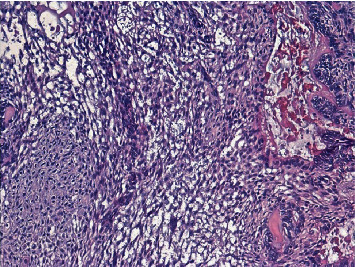
The tumor cells appear clear with eosinophilic cytoplasm, according to the differentiation H&E ×10.

**Figure 8:**
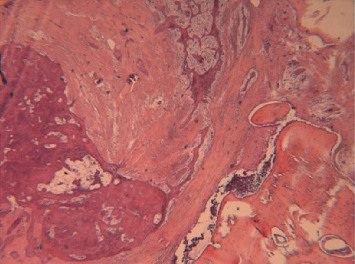
H&E ×25: mixed type of follicular and acanthomatous solid ameloblastoma.

**Figure 9:**
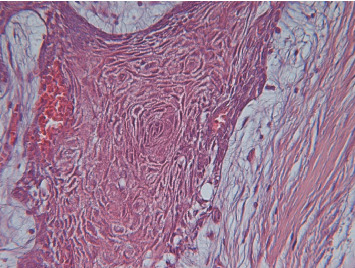
Detail from the previous histological preparation. H&E ×250: magnification of the acanthomatous component; Malpighian differentiation is highlighted by the presence of desmosomes.

Histological examination of the surgically excised tissue confirmed the tumor recurrence (Figures 6–9).

**Figure 10:**
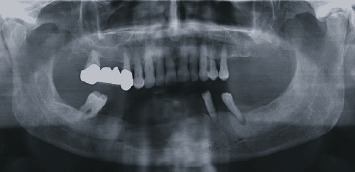
Four-year radiographic follow-up. No recurrence was detected.

No recurrence was observed at the radiographic 4-year follow-up (Figure 10), and no further diagnostic exams were performed due to the difficult clinical conditions of the patient who subsequently died due to other causes at the age of 83.

## 3. Discussion

The epidemiological features of the present case support those reported by the international literature regarding AA, despite the association of two different histo-types. Actually, the advanced age of the patient (78 years) is typical of the AA whose mean age at presentation (51 years) is more advanced than that of both the follicular (41 years) and the plexiform (39.1 years) subtypes [1]. Moreover, the mandibular canine-premolar localization of the present case corresponds to the generic ameloblastoma's predilection for the mandible (81%) and in particular to that of the AA for the mandibular incisor-canine area (42.8%), while the plexiform and follicular subtypes prefer both the molar area and the ascending branch (31.5%). No difference, on the other hand, was found in the literature with reference to gender and race [1, 10].

Although ameloblastomas with more than one epithelial hysto-type are not infrequent, mixed cases with acanthomatous hysto-type are rarely reported in the literature [12–14], and, to the best of the authors' knowledge, no cases of mixed ameloblastomas in which the acanthomatous component is so much represented, except for the present case, are reported in the international literature.

Pathogenesis of the coexistence of different hysto-types in the same tumor has not been explained yet. It is possible that it represents the different expression of the same neoformation process due to epithelial ameloblastic cells in different stages of maturation and morpho-differentiation—in relation to genetic mutations of their pathway of differentiation [15–17] or to different epigenetic or nongenetic time- and site-related stimuli [18]—or due to cells derived from different portions of the dental lamina, rather than the early or late association of different neoplasms or the conversion of one hysto-type into another, as already suggested in the past [19]. The genetic study of the various histological subtypes present in the mixed forms of ameloblastoma could provide useful information on their pathogenesis as well as allowing a targeted therapeutic strategy.

The clinical radiographic features of this case also support those described in the literature.

Actually, bone swelling seems to be the most common clinical sign of all types of ameloblastoma.

Other frequent events are pain, soft tissue ulceration, tooth eruption disorders, tooth mobility, and dislocation [1, 6, 8]. Dislocation was particularly evident in the present case, given the remarkable diastema between the involved teeth. Reichart et al. [1] already identified that AA caused cortical bone involvement more (50%) than all other types in the case of expansion of the involved bone segment, as in the present case.

All these clinical features, however, as well as the radiographic appearance, were not pathognomonic of ameloblastoma. Actually, differential diagnostic difficulties exist with several other noncystic osteolytic lesions of the mandible, since unilocular and multilocular aspects of the solid/multicystic forms seem to occur with approximately the same frequency [1, 5, 8].

The decision to first perform an excisional rather than an incisional biopsy of the lesion was made based on the following aspects: the diagnostic assumption of a benign neoformation due to the limited extension to the canine region in a subedentulous mandible; the lack of involvement of the nervous and vascular surrounding structures (mental bundle); the good radiographic peripheral demarcation, the radiographic appearance not pathognomonic of ameloblastoma; the hypothesis to resolve the pathological picture in a single surgical session, in view of the disability, with lack of mobility; the advanced age of the patient; and the notable distance of his home from the hospital. Actually, to the best of the authors' knowledge, there are no guidelines in the international literature on when to perform an excisional or incisional biopsy of jaw bone lesions. It therefore seems appropriate to obtain a precise histological diagnosis preoperatively using a preliminary incisional biopsy in the presence of an apparently benign osteolytic lesion of an uncertain nature, wide enough and/or involving important adjacent anatomical structures such as nerves, blood vessels, mucous membranes, and roots of many teeth, in order to perform a proper surgical planning, reducing the risk of recurrence caused by an incomplete intervention. Actually, the frequency of ameloblastoma recurrence, which may occur even many years after surgery, seems to depend on both the histological features of the tumor and the type of therapeutic approach used [1, 4, 6–8].

The extensive 1995 literature review by Reichart et al. [1] (3,677 cases) showed that tumors of follicular and plexiform subtypes resulted having a high frequency of recurrence (16.7% and 29.5%, respectively) compared to the acanthomatous, unicystic, and peripheral subtypes which, instead, appeared associated with a relatively lower recurrence rate (4.5%, 13.7%, and 9.1%, respectively).

However, the most recent study by Hong et al. [4] with 305 cases reported a 26.47% recurrence rate for AA, comprised between that of the follicular/granular cell subtypes and that of the plexiform/unicystic subtypes [5–7, 9]. The current WHO classification of odontogenic tumors [11] finally reports that the hystological type does not condition the prognosis, and it is therefore possible to argue, in the same way, that the presence of different histological types in the same tumor does not determine an evident improvement or worsening of the average tumor prognosis, and, probably, the different therapeutic approach is the most important factor in recurrence frequency which seems related to an uncompleted tumor removal [11], as happened in the present case, as well as to the tumor genetic profile, which were both found to be independent, statistically significant predictor factors of recurrence [17].

Since the simple enucleation or curettage of solid/multicystic ameloblastoma has 60-80% recurrence rate, especially in the presence of high-risk histologic variants such as the follicular one, lesion excision with an adequate safety margin is necessary [4, 7].

Histological sections showed tumor cells at a distance of 8 mm from the clinical and radiographic limit of the lesion, so the elimination of at least 1 cm of peripheral bone margin and the removal of at least 1 tissue plane surrounding the tumor when the soft tissues are involved seem to ensure the complete elimination of all possible peripheral tumor nuclei and may therefore significantly reduce the recurrence rates [4, 7].

The close clinical radiographic follow-up of the patient was therefore essential for early detection of recurrence and for its rapid treatment. Its small size compared to that of the initial mass also allowed to perform a much less aggressive or radical surgery, compared to what could have been in the first instance. Such a procedure allowed the removal of about 7-8 mm of clinically and radiographically healthy bone tissue, laterally and inferiorly to the lesion, and the simultaneous preservation of the lingual cortical bone integrity, in order to reduce surgical invasiveness and promote better tissue healing. The decision to conservatively treat the recurrence with a rim resection took into account its small size, histological type, and the advanced age and the clinical conditions of the patient. Actually, although neoplastic projections are able to spread easily through the marrow spaces of the spongy bone, it is unusual that the tumor tissue infiltrates the compact bone as well as the neural structures such as the inferior alveolar nerve [1, 4].

Finally, since the slow growth of these tumors can take many years (even 20 years) before a recurrence may be seen, although it most commonly appears 2-5 years later, the surgical site clinical radiographic follow-up is highly recommended for at least 10 years [4, 7, 9]. In the present case, follow-up was not possible because of the patient's death which occurred 5 years after surgery due to other causes.

## 4. Conclusions

Clinical and radiographic features of an early stage ameloblastoma can be not pathognomonic, so a preliminary histological diagnosis, through an incisional biopsy, seems highly indicated. During surgery, the preservation of the lingual cortical is advisable unless it is not clearly compromised by the tumor, in order to preserve bone continuity. Acanthomatous ameloblastoma is not frequent, but its complete excision, with adequate peripheral safety margin, is of paramount importance in reducing the risk of recurrence.
